# Preoperative subjective impairments in language and memory in brain tumor patients

**DOI:** 10.3389/fonc.2024.1475860

**Published:** 2024-10-29

**Authors:** Lena Rybka, Roel Jonkers, Milena Burzlaff, Tizian Rosenstock, Peter Vajkoczy, Thomas Picht, Katharina Faust, Adrià Rofes

**Affiliations:** ^1^ Center for Language and Cognition, University of Groningen, Groningen, Netherlands; ^2^ Research School of Behavioural and Cognitive Neurosciences (BCN), University of Groningen, Groningen, Netherlands; ^3^ Department of Neurosurgery, Charité – Universitätsmedizin Berlin, Corporate Member of Freie Universität Berlin, Humboldt-Universität zu Berlin, and Berlin Institute of Health, Berlin, Germany; ^4^ Berlin School of Mind and Brain, Humboldt University, Berlin, Germany; ^5^ Berlin Institute of Health at Charité – Universitätsmedizin Berlin, BIH Biomedical Innovation Academy, BIH Charité Digital Clinician Scientist Program, Charité, Berlin, Germany; ^6^ Cluster of Excellence: “Matters of Activity. Image Space Material”, Humboldt University, Berlin, Germany

**Keywords:** brain tumor, language, memory, subjective deficits, quality of life

## Abstract

**Background:**

Subjective reports can reveal relevant information regarding the nature of the impairment of brain tumor patients, unveiling potential gaps in current assessment practices. The co-occurrence of language and memory impairments has been previously reported, albeit scarcely. The aim of this study is therefore to understand the co-occurrence of subjective language and memory complaints in the preoperative state of brain tumor patients and its impact on Quality of Life (QoL).

**Methods:**

31 brain tumor patients (12 LGG, 19 HGG) underwent a semi-structured interview to assess subjective complaints of language deficits, co-occurrences between language and memory dysfunction, and changes in QoL. Group and subgroup analyses were conducted to provide general and tumor grade specific data.

**Results:**

48.4% of patients mentioned co-occurrence of language and memory impairments in reading, writing, and conversation. The HGG group reported co-occurrences in all three of these (reading: 31.6%; writing: 21.1%; conversation: 26.3%), while the LGG only described co-occurrences in reading (25%) and conversation (8.3%), although these were not statistically significant. All patients with co-occurring language and memory deficits reported these to be linked to reduced QoL (48.4%). In patients with an HGG, this number was slightly higher (52.6%) than in patients with an LGG (41.7%).

**Conclusion:**

Language impairments co-occur with memory dysfunction as perceived in patients’ daily life. Patients see these impairments as affecting their quality of life. Further attention to dedicated language and memory tasks seems necessary.

## 1 Introduction

Evaluating multiple language functions is becoming common practice in the assessment of brain tumor patients (1–3). However, detailed evaluations of short-term and working memory (henceforth “memory”) are less common [e.g. (4)]. More importantly, difficulties with memory have been previously reported (4–8) and can co-occur with language deficits (9–11). This relation between language and memory has been discussed in both healthy [for review see (12)] and clinical populations [e.g. (13–16)], suggesting, for example, that memory capacity is relevant for language production and comprehension as it relies on encoding, maintenance, recall, and manipulation of information [e.g. (17)]. Consequently, memory impairments have been associated with issues in sentence processing, reading comprehension, writing, and more generally with conversation abilities (4, 9, 11, 18–20). To illustrate, complex sentences may require a higher memory load than simpler sentences (21, 22). Damage of memory function can hence limit the ability to comprehend linguistically complex structures. Despite the co-occurrence between language and memory impairments, difficulties with memory and language are not consistently reported. Potential factors for this inconsistency may relate to patient selection (e.g., focusing on a specific tumor type, location, surgical intervention), or the use of different assessment protocols which include different tasks across centers (3, 10, 11, 23). For example, patients with tumors that grow particularly fast (e.g., high grade gliomas, HGGs) may be more severely affected in language and memory than individuals with slower growing tumors (e.g., low grade gliomas, LGGs; 4, 6, 24). Regarding protocols, survey work indicates that object naming and fluency tasks are typically used to assess brain tumor patients (2). However, other materials to assess memory are used more sparsely (3). Additionally, the tasks typically administered have often been standardized to assess stroke patients, where the sudden disease onset may cause more pronounced symptoms than in brain tumor patients (25).

The study of subjective complaints may provide complementary information to dedicated protocols, possibly unveiling potential gaps in current assessment practices. To assess subjective complaints, studies commonly use standardized Quality of Life (QoL) questionnaires. Examples are the EORTC-QLQ-C30 and BN20 (26) which address a wide range of topics to assess patient’s well-being, symptom burden, and symptom management (27). However, these questionnaires often provide limited questions related to language and memory. To illustrate, the EORTC-QLQ30 and BN20 only include four questions relating to language (1x difficulty to read, 1x word finding difficulties, 1x difficulties to speak, 1x difficulty to communicate thoughts) out of a total of fifty questions. Memory disturbances are only reflected in one question.

Semi-structured interviews offer an alternative approach to these questionnaires. Previous studies employing this method highlighted the importance of language and memory and their co-occurrence for a wide variety of QoL-related factors, including work ability [e.g. (18, 28–30)], social participation and psychological distress [e.g. (31–33)]. For instance, patients with brain tumors reported a negative influence of memory disturbances on their communicative abilities that were perceived as limiting them in recollecting thoughts, the conversational content or the ongoing topic (18, 28, 34). These findings may suggest that good language performance does not only rely on an intact language system but also on other neurocognitive functions, such as memory [for discussion see (35–37)]. These findings cannot be validated using standardized questionnaires which highlights the added value of patient interviews.

Of particular interest for the study of the tumor impact itself on both language and memory functions and the consequences for QoL, preoperative assessments in brain tumor patients are indispensable, and also serve as a baseline for follow-up examinations [e.g. (2)], where treatment effects such as surgery and adjuvant therapies may have triggered (further) neurocognitive decline (1, 38, 40). Additionally, a preoperative baseline is crucial to determine the longitudinal trajectory of neurocognitive changes which can provide valuable information on the possibly differential recovery patterns across various neurocognitive functions. Indeed, some studies suggest that memory may recover more poorly after brain tumor surgery compared to language (40). Considering the above-mentioned detrimental effects of such long-term deficits on QoL, such as work ability (28, 30), these findings suggest that memory may be a crucial neurocognitive function to preserve, and requires preoperative assessment. Despite this relevance, preoperative deficits have not been as widely studied as postoperative neurocognitive deficits (11, 39, 41, 42), although patients with reduced QoL at baseline may also report QoL limitations at later stages (43). This is particularly true for qualitative studies assessing subjective neurocognitive complaints in brain tumor patients [e.g. (29, 44)]. Another aspect to consider is that these studies usually do not report the prevalence of these subjective deficits or differentiate patients based on tumor characteristics [e.g. (32)], leaving an uncertainty as to the relevance for the overall population of brain tumor patients or specific subgroups (e.g., patients with LGGs or HGGs). Differences across different tumor groups, especially in relation to tumor grades, have been previously reported, including language and memory function (6, 23, 45), mainly based on neuropsychological tests. Understanding whether this may translate to subjective dysfunctions, is necessary to improve patient consultation or neurorehabilitative measures.

The scarce literature available on preoperative subjective complaints supports the notion that language and memory dysfunction are present before treatment [e.g. (41, 46)] and limit QoL [e.g. (32)]. Considering that these studies rarely contrast patients with different tumor grades, the influence of these tumor grades on perceived preoperative impairments remains unclear. Taken together, the current information provided by these qualitative studies using interviews to assess subjective deficits, mainly hints at subjective changes particularly perceived in communication, with some observations of co-occurring memory deficits. Other difficulties, such as struggles with more specific aspects of language such as writing or reading, are less frequently studied, causing a possible gap in the current literature.

### 1.1 Aims and predictions

We aim to understand the preoperative co-occurrence of subjective language and memory deficits in individuals with brain tumors and their relation to QoL. We will answer the following research questions:

1. Do language and memory deficits co-occur in preoperative subjective reports of brain tumor patients? If so, which are the most frequently reported language modalities (e.g., conversation, reading, writing) of this co-occurrence?
2. Are these deficits related to a perceived decline in QoL?
3. Do patients with LGGs differ from those with HGGs in their subjective reports?

We hypothesize that disturbances in memory and language will co-occur. Furthermore, we expect that most co-occurrences may be perceived during conversation, and that language and memory deficits contribute to reduced perceived QoL in patients. Finally, HGG patients will present with more difficulties and a higher rate of co-occurrence compared to LGG patients.

## 2 Materials and methods

### 2.1 Participants

Thirty-one patients with gliomas (mean age = 41.19, SD = 1.76, range = 22-61, male = 11, female = 20) were included. Twelve patients had a LGG and 19 had a HGG. Eligible patients were screened based on the diagnosis of a presumed glioma at the neurosurgical department of the Charité Universitätsmedizin Berlin from January 2023 until June 2024. Initial diagnosis was based on MRI scans, medical history, and physical exam. Data on demographic and tumor characteristics can be found in **Table 1**.

**Table 1:** Demographic and tumor characteristics.

Patient	Age	Gender	Tumor diagnosis	WHO grade	Hemisphere	Location
P1	55	female	oligodendroglioma	3	left	parietal
P2	37	female	pediatric diffuse HGG	4	left	parietal
P3	57	female	diffuse LGG	1	left	temporal
P4	24	male	astrocytoma	2	left	insular
P5	47	female	glioblastoma	4	left	parietal
P6	52	male	glioblastoma	4	left	temporal
P7	25	female	astrocytoma	2	left	frontal
P8	45	female	glioblastoma	4	left	temporal
P9	27	male	oligodendroglioma	2	right	frontal
P10	34	male	astrocytoma	3	left	temporal
P11	61	female	glioblastoma	4	left	temporal
P12	38	male	oligodendroglioma	2	left	frontal
P13	51	female	diffuse LGG¹	1/2	left	frontal
P14	45	male	glioblastoma	4	left	temporal
P15	33	female	astrocytoma	1	left	temporal
P16	61	female	astrocytoma	4	right	temporal
P17	36	male	astrocytoma	2	left	frontal
P18	35	female	astrocytoma	4	left	insular
P19	59	female	astrocytoma	3	left	parietal
P20	37	female	glioblastoma	4	right	temporal
P21	36	male	oligodendroglioma	3	left	parietal
P22	37	female	oligodendroglioma	2	left	temporal
P23	22	female	astrocytoma	3	left	temporal
P24	37	male	oligodendroglioma	3	right	frontal
P25	46	female	oligodendroglioma	2	left	frontal
P26	38	female	astrocytoma	3	right	parietal
P27	38	female	astrocytoma	3	right	insular
P28	39	male	astrocytoma	2	left	frontal
P29	51	male	astrocytoma	3	left	insular
P30	42	female	astrocytoma	3	left	frontal
P31	31	female	astrocytoma	2	right	frontal

¹no further histopathology available.

The inclusion criteria for this study were: having pathological results confirming glioma diagnosis; being a native German speaker; and presenting with no severe language deficits, rendering the administration of the semi-structured interview impossible. Potential patients were then contacted via phone to provide information about the study and to ensure that inclusion criteria were met. Patients who were scheduled for surgery on short notice were provided with the study information during their hospital stay at least two days prior surgery. Ethical approval for this study was obtained from the Ethical Review Board of the Clinic (no. EA1/050/23). All patients consented to participate and signed a consent form.

### 2.2 Materials

A guide for the semi-structured interview was designed to ensure that all patients underwent the same questions and to avoid question omittance. The questions were based on findings from previous studies and deficits reported in admission and dismissal letters from the clinic. Importantly, the questions were pre-arranged into six topics to ease interview conduction as those topics evolve around the way patients use language in their daily lives, as well as to combine all relevant information for each topic for later analysis. Furthermore, if topics are used during patient consultation and screening that focus on easily identifiable topics based on daily life activities, identifying possible deficits may be facilitated for both the clinician and the patient. Topics included frequently observed language deficits (e.g., lexical retrieval deficits), topics revolving around the use of language in patients’ daily lives (e.g., reading, writing, conversation), and QoL (e.g., work ability, family and social life, leisure activities). After a general question about each topic (“Have you perceived changes in writing?”), follow-up questions were posed (“Do you have problems constructing longer or more complex sentences?”). These follow-up questions were designed to capture more specific and subtle changes within each topic. To determine possible co-occurrences, the authors adhered to indicators in patient reports relating to memory, such as (problems with) recall, retention, or storage of information.

During the interview, the interviewer (LR) was allowed to ask additional questions to accommodate the patient’s answers and ask for clarifications or examples, when needed. The interviewer ensured that patients had enough time to elaborate, repeat or reformulate questions, if needed. Therefore, no time limit was set for the interview. The duration of the interview questions varied between 5 and 32 minutes with a mean time of 14 minutes (SD= 9). The interview was conducted via phone call (N=5), online meeting (N=8), or in person in the clinic (N=18).

The full interview also comprised questions relating to socio-emotional functioning. These will not be reported in the current study, as they are deemed out of scope. Furthermore, patients reported a high variety of language deficits. In order to address these data in a suitable manner, especially with regard to the complexity and volume, another publication will be devoted. Here, we will report on the topics that allowed us to concentrate on the co-occurrence of language and memory deficits.

Examples of the questions are listed in **Table 2** and the full series of questions included in the semi-structured interview can be found in the **Supplementary Materials**.

**Table 2:** Examples of interview questions for language (ex. 1-3) and QoL (ex. 4-7)¹.

1. Have you perceived changes in writing?
1. Do you write slower (and if, why)?
2. Do you have problems finding the right words?
3. Do you have problems compiling the text?
4. Do you have difficulties constructing sentences when reading?
2. Have you perceived changes in reading?
1. Do you read slower (and if, why)?
2. Do you have problems understanding what you read?
3. Do you have difficulties understanding sentences when reading?
3. Have you perceived changes in conversation?
1. Do you more frequently struggle to understand what another person is saying?
2. Do you have problems participating in debates?
3. Do you have problems following or understanding what others say?
4. Do you have difficulties in pursuing leisure activities?
1. If so, why? Which factors contribute to the inability to do so?
2. Have you adopted new leisure activities as a replacement? If so, why are these easier?
5. Have you perceived changes to your family life?
1. If so, why? Which factors contribute to these changes?
2. Do you perceive these changes as negative?
6. Have you perceived changes to your social life?
1. If so, why? Which factors contribute to these changes?
2. Do you perceive these changes as negative?
7. Have you perceived changes in your ability to work?
1. If so, why? Which factors contribute to these changes?
2. What has exactly changed?
3. Do you perceive these changes as negative?

¹All questions are first followed by the prompt to give an example in case the patient does not immediately provide an example. For readability purposes, this is omitted here for every question.

### 2.3 Data collection

The interviews were audio-recorded, transcribed verbatim using an automatic speech recognition system [e.g., Whisper (47),], and manually checked by a student assistant (MB) who is a native German speaker. Properties of speech, such as crying or laughing, were not transcribed, as they were not deemed relevant for the purpose of this study. After manual correction, LR extracted the relevant passages from the transcription, noted the presence or absence of a language deficit, and whether the participants reported a co-occurrence of language and memory deficits. These passages also served as citations to illustrate the impact of present deficits on the patients’ lives. Based on this data, we identified those topics where patients most commonly perceived a co-occurrence of language and memory. Any doubts regarding the co-occurrence of language and memory deficits were checked with the senior author (AR).

### 2.4 Analyses

Descriptive statistics were performed to report frequency of perceived deficits. We conducted a group-level analysis that included the full cohort of this study, followed by a subgroup analysis to assess whether individuals with an LGG and those with an HGG differ from one another. **Table 3** presents examples of this process (examples translated from German into English). Fisher’s exact test was used to determine significant association between subgroups (LGG and HGG) and subjective co-occurrence of language and memory deficits.

**Table 3:** Examples of transcription extracts, topics and specifications.

Extract from transcript	Language deficit present?	Co-occurrence?	Topic
When I try to write, I need to think a lot about what I want to write. First, I know what I want to write, then I suddenly lose my train of thought and do not know what I wanted to write. If I then remember later on, I often cannot find the right word, or I forget what I was writing in the middle of the sentence. It sounds stupid, but sometimes I forget what I started to write at the end of a sentence.	Yes	Yes	Writing
When I talk to my partner, I need more time than before because I cannot think of the word I want to say. [ … ] But I know what I want to say and can remember everything like before, it just takes longer because I need to think more.	Yes	No	Conversation

## 3 Results

### 3.1 Language and memory co-occurrences

**Figure 1:**
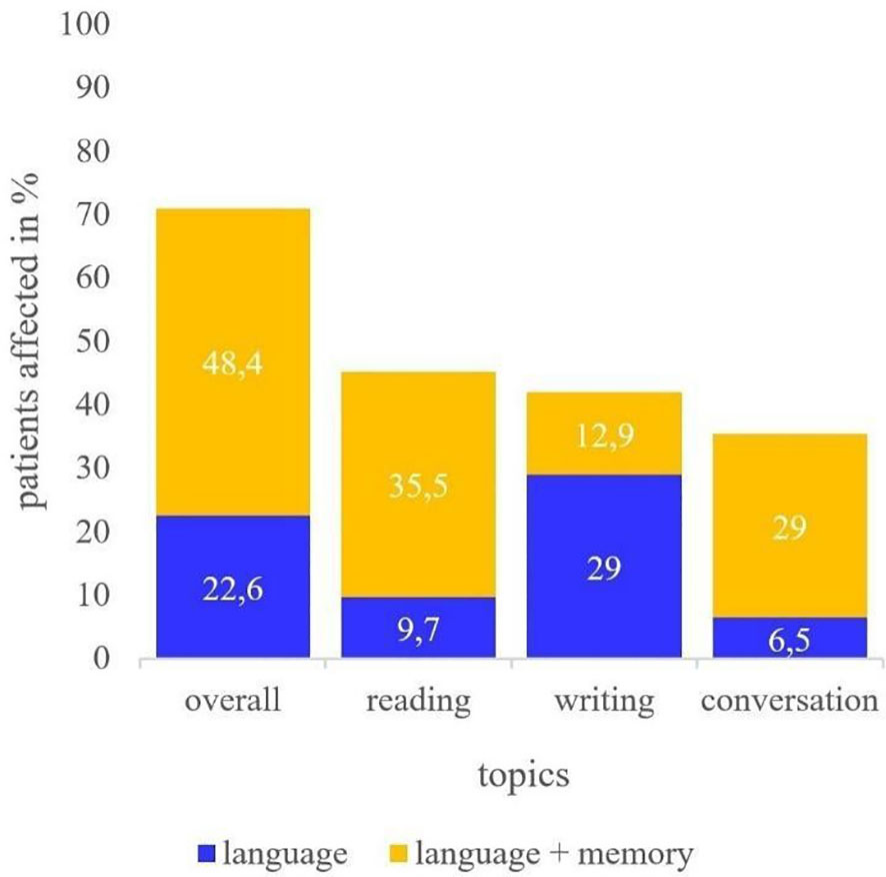
Deficits reported in reading, writing, and conversation. Report of patients perceiving language deficits only (blue) or both language and memory deficits (yellow).

Co-occurrences of language and memory impairments were reported in the following three topics: reading, writing, and conversation. In total, 71% (n=22) of the participants reported difficulties within these three topics, with 48.4% (n=15) reporting a co-occurrence of language and memory deficits. The greatest number of co-occurrences was observed in reading (35.5%; n=14), followed by conversation (29%; n=11). Co-occurrences in writing were less frequently reported (12.9%; n=4; see **Figure 1**).

In reading, language deficits involved problems relating to dysgraphia and lexical retrieval, while the simultaneous presence of language and memory impairments was noted in, for example, the maintenance of information from sentences or whole passages [1,2].

[1] I recently bought a new book. [ … ] I did not get along with it. I did not understand the language. [Long sentences] are difficult to understand the text, I just cannot keep [them] in mind.

[2] Even if I understand everything in terms of content, even if I look at it at short intervals, and can still understand the content of the sentence, but I can no longer keep it in the same way.

Difficulties in writing were furthermore reported due to impairments in language, such as lexical retrieval [3], but also due to co-occurring memory dysfunction [4].

[3] When a word is missing, I especially notice that when I am writing. Then I cannot recall, for five seconds, how to write a simple word. And after the five seconds, it is immediately back again.

[4] And I often have to think about how do I write this now and then what did I want to write?

In conversation, patients described similar problems relating to an inability to process and recall information from complex or long sentences, as well as maintaining the on-going topic [5-7].

[5] I [sometimes] do not even understand what [someone] is saying to me. I really panicked because I thought all the words in my head did not come together to form a sentence. She repeated that to me 30 times and I was like, I do not understand. And I could not, I could not say anything. [ … ] So, I have moments like that every now and then.

[6] What do I want to say and which words can I logically compile for somehow making up a sentence to produce?

[7] [When I listen to someone] the long sentences are bothering me, [it is] the length and the information density.

### 3.2 Language, memory, and quality of life

A reduced QoL was reported by 61.3% (n=19) of the participants. Of these, 79% (n=15; 48.4% of the overall cohort) reported their limitations in QoL to be associated with the impairments in language and memory. 10.5% (n=2; 6.5% of the overall cohort) reported reduced QoL due to language deficits only. The remaining participants (10.5%, n=2; 6.5% of the overall cohort) related these to worries and anxiety.

Changes in work ability were noticed in 12 of 31 patients (38.7%) in relation to cognitive deficits [8-11].

[8] I could not recall everyday words that I need in the context of work. There was a meeting and I could not think of the word “brainstorming”. That is a standard, being able to do this. [ … ] That happens frequently.

[9] I have a lot to do with processes and its steps. And processing steps also means that I need to start somewhere, opening a folder where the file is that I will need. I need to access this file and need the folder in which this information is. This has become increasingly more difficult [for 5 years]. [ … ] And now I have written down, I get to that folder because I need that [file].

[10] Then a few things with work colleagues. And then there were conversations again [that I could not understand], and conversations that had a little to do with legal data. And of course, you have to concentrate carefully, and remember. And there are a few things that I have to ask again.

[11] And [ … ] it has been much, much more strenuous for me to speak like that, to have intensive conversations. I can no longer give a speech, which I could do before. And, of course, I have always been a bit more careful about the language. But at the moment it is extremely difficult. Where do I have the common thread, where do I have to start?

Other commonly reported QoL complaints were due to increased worries, such as relating to epilepsy onset, neurocognitive deficits or surgery, affecting family life and leisure activities [12-16].

[12] I initially had a strong fear of an epileptic seizure, so I have not dared going outside alone. So, I had a friend with me a lot. It got better because I have not had another seizure or, when [I could feel it approaching], I have developed my “calm-down-methods” and noticed that they worked.

[13] I do not leave the area I am living in alone. [It is] too much. The seizures in my head, that I get a new seizure. Hence, I do not leave [my area] alone.

[14] Of course you are worried. Definitely, yes. This is true for every area, especially concerning family and when it comes to the children. Well, to be completely honest, when I saw the two [children] last time, I cried bitterly.

[15] I have taken care of many things [in case anything happens], wrote the health care proxy for my brother, insurances everything clarified, financially everything clarified, made videos [ … ] for every single person. Today, as I said, I have written a letter to my son, today I want to write another letter to my daughter today. Because, I really worry about what is going to happen in the future, when I am not there anymore. That is a great burden. What is also a burden in addition is that I was always the strong part, who kept the family together. [ … ] And if I do not have the opportunity anymore because I cannot walk properly, because I cannot express myself properly, yes, that worries me a lot [ … ]. And that I cannot be the father that I am and want to be.

[16] So, I have had great worries now and I am simply nervous and glad, when the surgery is over and the tumor is removed, because I worry due to my family history [of having brain tumors].

Besides family life, also social interaction in general was affected in those patients, partly due to their cognitive deficits [17-21].

[17] Now it is the case that I cannot think of arguments. And then I prefer to break off such a discussion because I then just get bogged down in it and then I get upset. And for example, my [partner] then thinks that I am upset with her. And then it turns into a fight, which of course I do not want. [ … ] But I [cannot recall the arguments] at the moment.

[18] I then have to weigh up what is more important to me at that moment. Be it now when I meet for coffee and I know there will be an interesting event in the evening. How early can I have coffee? Can I estimate in advance whether it is a casual [conversation] or is it a somewhat more in-depth conversation? [ … ] My social life also suffers a bit [ … ] and that really gets me down.

[19] I don’t exchange ideas with several of them because there is no kind of understanding. [ … ] It [is] a bit fileted and not everything is discussed with everyone, with a few, but rather divided up a bit.

[20] Otherwise, I was often the one who could talk endlessly, without any problems, and also make people feel comfortable, so it is not like I just speak alone, but I also involve people. But now dialogues are much more difficult for me to follow and keep up with at some point and then not to leave at some point.

[21] And actually I wrote something and I thanked them. And the other one somehow replied that she was so disappointed in me. And I thought, what did I write to her then? I will have to check that again later. But I already wrote in response “I said I liked to, that I love you like the [other friends]. What was spelled wrong? I will have to take a look at that.

### 3.3 Differences between LGG and HGG

**Figure 2:**
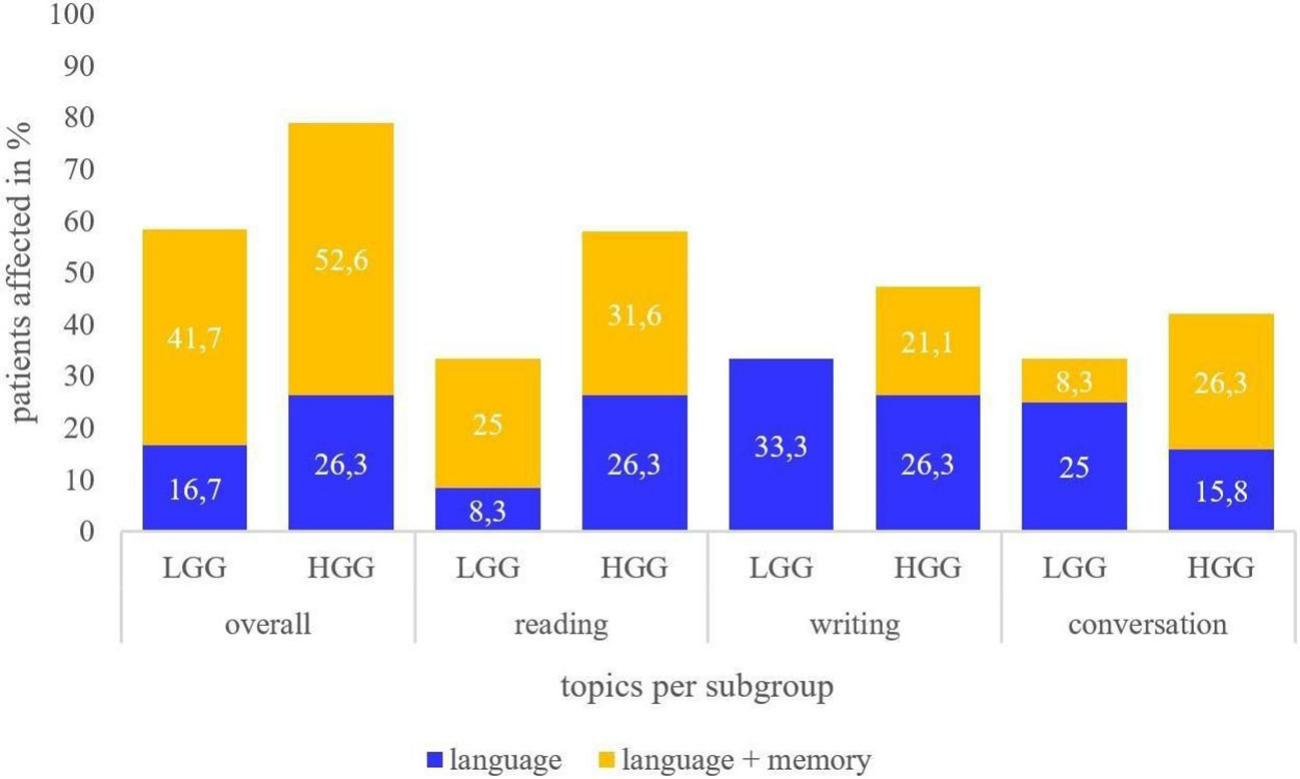
Deficits reported in reading, writing, and conversation in LGG and HGG subgroups. Report of patients perceiving language deficits only (blue) or both language and memory deficits (yellow).

In reading, writing, and conversation, further differences between both groups were observed (see **Figure 2**).

HGG patients reported a higher number of co-occurrences of language and memory dysfunction compared to LGG patients (52.6%; n=10 vs. 41.7%; n=5). Importantly, HGG patients perceived more co-occurrences of language and memory in reading and conversation than language deficits only (reading: 31.6% vs. 26.3%; conversation: 26.3% and 15.8% respectively). Those with a LGG also described more co-occurrences in reading than language deficits only, as well (25% vs. 8.3%), but more language deficits than co-occurrences in conversation (25% vs. 8.3%). In writing, the LGG group did not report any co-occurrences. Reading, hence, seems to be most frequently affected by co-occurrences in language and memory difficulties in the full cohort, while co-occurrences in writing were only observed in the HGG group. These differences are, however, not statistically significant (p= >.05, two-tailed).

In relation to patients’ QoL, 58.3% (n=7) of the LGG group reported reduced QoL, while 63.2% (n=12) of those from the HGG group perceived a reduced QoL. All patients with co-occurring language and memory deficits reported reduced QoL. For the LGG group, this comprises 41.7% (n=5) of the group, and 52.6% (n=10) of the HGG group. Of the LGG patients, 16.7% (n=2) described a decline in QoL due to language deficits while they did not report co-occurring memory deficits. 10.5% (n=2) of the HGG group related their reduced QoL to their increased worries and anxiety. For HGG patients, work ability was the main QoL factor to be affected (HGG: 52.6%; n=10), while those with a LGG were similarly affected across work ability, family and social life (25% each; n=3).

## 4 Discussion

In the present study, we set out to assess the co-occurrence of language and memory deficits as subjectively perceived in the preoperative stage of a group of glioma patients. Analysis revealed co-occurrence of language and memory deficits in reading, conversation, and writing. While conversation and communicative abilities have been reported in previous qualitative work [e.g. (34)], the co-occurrence of language and working memory in these other domains has been seldom reported (28). To the best of our knowledge, this is the first study to explicitly focus on the co-occurrence of language and memory deficits in individuals with brain tumors.

In line with our hypotheses, individuals with brain tumors reported co-occurrences of language and memory deficits. These co-occurrences were reported by roughly 48% of our participants. Contrary to our predictions and the most frequently examined topic in the interview-based literature [e.g. (34)], conversation was not the most commonly reported topic where language and memory deficits were perceived to co-occur. Instead, co-occurrences were also frequently reported in reading (reading: 35.5%; conversation: 29%). This is an interesting finding, as reading has not yet gained as much attention in the scientific literature, as, for example, conversation. It may therefore be a relevant topic to further observe in this population considering the number of patients reporting difficulties in reading in their daily lives. Writing was the topic least affected by such co-occurrences (12.9%). Available preoperative subjective data by other authors also indicates both language and memory deficits and a negative impact on QoL [e.g. (32, 46)]. Patients in the study by Walter et al. (32) reported the greatest deficits in reading, similar to our findings, and further described deficits in writing, in addition to communicative limitations. These are three relevant topics also identified in this study. Whether the subjective complaints in Walter et al. (32) are partly related to co-occurring memory deficits cannot be determined, as this type of information was not reported. It can only be noted here, that their patient cohort also reported memory deficits.

It is interesting to see that postoperative findings in the current literature are in line with our preoperative data, as they also describe language and memory co-occurrences and the reduced QoL caused by these deficits [e.g. (18, 28)]. These studies report similar patient perceptions, such as forgetting the topic of an ongoing conversation or recalling content of conversations. Despite similar patient reports in our preoperative study, the comparison may not be as reliable considering the possible impact of treatment and adjuvant therapies in any postoperative cohorts. If we consult findings from studies employing quantitative methods to complement these results, we find that those are also in line with the present results [e.g. (9, 10, 45)] and may provide further explanations on the nature of this co-occurrence. These studies may suggest that memory function is crucial for both language comprehension and production (17, 37), including sentence processing. Patients in the present study reported, for example, subjective deficits at the sentence level. Hence, a reduced memory capacity may have partly affected language processing in production (writing, conversation) and comprehension (reading, conversation) alongside the language deficits. It seems, however, that co-occurrences of memory and language deficits are not found in all patients, requiring further studying of the precise mechanisms of these subjective impairments.

Confirming our hypothesis, language and memory co-occurrences were mentioned in relation with reduced QoL in the patients affected by these deficits. Besides work limitations, worries and changes in social and family life were related to poor QoL. This is in line with previous literature on QoL [e.g. (48, 49)], particularly in relation to issues such as return to work (20, 28, 29). Additionally, and even though this is not the main topic of the current paper, we also observed associations of language and memory complaints with increased worry and fear about the future and about social participation, which was also observed previously (18, 31, 49, 50). Consequently, deficits in language and memory have a widespread impact on many facets of QoL, and this study contributes preoperative data highlighting that QoL is already impaired preoperatively. Capturing this impact preoperatively is important to develop strategies about possible support a patient may need, including the development and deployment of new assessment tools, neurorehabilitative measures (pre- and rehabilitation), or psychooncological care for those with difficulties in their family and social life or psychological burden, especially if reduced QoL before treatment is indicative of QoL limitations at later stages (43).

Furthermore, based on the observation that co-occurring language and memory deficits were perceived as more detrimental to subjective QoL compared to those with only language dysfunction, memory may be an important neurocognitive function whose preservation is crucial to a wide variety of QoL areas, for example, regarding the ability to work which is in line with previous findings (18, 28, 30). If additionally, memory should indeed recover more poorly than language, as indicated by previous studies (40), and if this impairment causes a negative impact on QoL, as reported in this study but also suggested by previous literature (28, 34), memory may be relevant to patients’ daily lives and activities, and therefore an aspect relevant to preserve. Future longitudinal studies that include such preoperative assessments (and re-assessments) at different timepoints after surgery, seem needed to determine whether memory, in addition to language, is a function that recovers poorly and therefore requires dedicated assessment perioperatively and longitudinally.

We also hypothesized that patients with a HGG would present with a higher rate of co-occurring language and memory deficits compared to those with an LGG, which could not be statistically confirmed. Consequently, these findings are not in line with previous studies that report a higher prevalence of deficits in patients with a HGG compared to those with a LGG, especially from objective testing [e.g. (4, 6, 24)]. A comparison to previous interview data can scarcely be drawn due to the limited number of studies using this method, as those studies often did not assess differences in relation to tumor characteristics, such as tumor grade [e.g. (32, 46)] or assessed only LGG patients (49). For LGG, Antonsson et al. (49) reported 8.7% of their cohort to have difficulties in conversation, which is a lower rate than in the LGG group reported in the present study (33.3%). This may be explained by the volume and details in questions we used in our study. In consideration of these non-significant differences across subgroups, it may be suggested that language and memory co-occurrence may be of importance to brain tumor patients in general and across different topics and activities, with negative impact on QoL. This may be stressed by the observation that all LGG and HGG patients who reported co-occurring language and memory deficits reported a reduction of QoL. It needs to be highlighted here that again the professional life was particularly affected in HGG patients, with an equal limitation of LGG patients in their professional, social, and family life.

The importance of language and memory co-occurrence may require a more rigorous assessment of memory. Further work may be needed to adapt current neuropsychological test batteries to the needs and often rather mild symptoms observed in brain tumor patients, and accompany these assessments with QoL measures to determine whether a closer examination of memory in addition to language improves. This may include return to work after surgery considering limitations in professional life patients of this study already had preoperatively. If future studies have similar findings as to the relevance of memory, especially in case of deterioration after surgery, assessing memory in awake surgery may be a relevant addition to preserve this function. To date, memory and language assessments may be recommended for both HGG and LGG patients.

### 4.1 Limitations and future directions

The study included a heterogeneous group. Although this was intended to determine the prevalence in the overall glioma population, demographic and tumor characteristics, including tumor location, and infiltration of white matter tracts, may differentially contribute to the subjective deficits assessed in this study [e.g. (8, 51)]. This information may need to be addressed in a future study with a greater cohort. Indeed, the present cohort is rather small, especially for the LGG group (n = 12), so we should proceed with caution as these findings may not be representative of the whole population, but rather serve as an explorative study.

Even though the questions we used are partly based on previous work (18, 28, 34), it is possible that some of the difficulties mentioned also relate to psychological effects such as worries and distress, which we did not directly account for (33). Further work could add questions regarding stress and anxiety, along with the questions we proposed [e.g. (50)]. It needs to be mentioned that the responses may indicate that other functions may also contribute to deficits in language abilities, such as reduced processing speed or attentional deficits. This will require further analysis.

The high prevalence of perceived language and memory co-occurrence may also be related to the ability to create compensation mechanisms. Patients may find it easier to compensate for language deficits (34), while compensation mechanisms for memory deficits may be perceived as more effortful, for example, they may require taking further actions, such as taking notes (46, 52). Therefore, difficulties with memory (vs. language) may be perceived as triggering greater limitations in QoL in those affected by both language and memory deficits. Differences in compensation strategies in language and memory impairments may therefore deserve attention in future studies.

Furthermore, the analysis in this study focused whether or not patients perceived memory deficits. Consequently, no strict differentiation of the nature of the memory disturbance (e.g., short-term or working memory) was made. The focus of this paper is on the added value of asking additional questions relating to memory and language as perceived subjectively by patients in an interview setting, as can be done, for example, by clinicians during patient consultation. Here, questions relating to patients’ daily activities was deemed to be easier understandable by the patient than questions relating to specific processing steps. An analysis using linguistic or neuropsychological models may supplement such patient-focused questions, and provide information on, for example, tasks that may be necessary to capture these subjective deficits. This was, however, considered out of scope for the present study.

Besides these limitations, the results from these semi-structured interviews provide relevant information on possible deficits and their relationship with QoL. Subjective data as provided by such interviews seems to be a powerful complement to neurocognitive assessments and questionnaires, as these alone may not provide sufficient information [e.g. (7–10, 23, 53)]. From a clinical perspective, these findings may be of further use: the topics defined in the questionnaire seem to yield various additional insights into patients’ deficits, which may make these useful and informative during patient consultation, but also to support the choice of neuropsychological tasks for patient assessment or during awake brain surgeries, and possible neurorehabilitative measures. Furthermore, a more exhaustive memory assessment in addition to language tasks may allow us to objectivize these findings.

## 5 Conclusion

Almost half of our sample report co-occurring problems in language and memory, when asked for preoperative difficulties using a semi-structured interview. These problems are particularly perceived in reading, writing, and conversation. Patients who report problems in both language and memory more frequently report limitations in QoL, than patients that report problems in language only. Further attention to the study of memory in this population seems granted.

## Data Availability

The raw data supporting the conclusions of this article will be made available by the authors, without undue reservation.
